# Copper influence on bank vole’s (*Myodes glareolus*) sexual behavior

**DOI:** 10.1007/s10646-018-1902-z

**Published:** 2018-02-02

**Authors:** Agata Miska-Schramm, Joanna Kapusta, Małgorzata Kruczek

**Affiliations:** Institute of Environmental Sciences, Jagiellonian University, Gronostajowa 7, 30-387 Krakow, Poland

**Keywords:** Copper, Bank vole, Sexual behavior, Preference test

## Abstract

The impact of human activity on the environment has led to a steady increase of the amounts of copper in the ecosystems. This element accumulates in plants and water, potentially exposing rodents to its harmful effects. In industrial districts, a decrease in the density of small rodent populations has been observed. This decline may be caused by many factors, including mortality, decreased fertility, or impaired sexual behavior. The decline in the reproductive abilities of small rodents after copper exposure was demonstrated in our previous work (Miska-Schramm A, Kruczek M, Kapusta J, *Ecotoxicology* 23:1546–1554, 2014). The aim of the presented research was to determine how copper administered at concentrations similar to those recorded in industrial districts (Cu I-150 mg/kg, Cu II-600 mg/kg, C-control) affects the sexual behavior of small rodents. The model species was the bank vole (*Myodes glareolus*). The behavior and vocalizations of male-female pairs were recorded during open-field tests: ♂C vs. ♀C; ♂Cu I vs. ♀C; ♂Cu II vs. ♀C while in preference tests, female behavior was assessed in the following combinations: ♀C vs. ♂C & ♂Cu I; ♀C vs. ♂C & ♂Cu II. In the presented work, we show that copper decreased the males’ sexual attractiveness. Females showed suppressed preference towards males treated with 600 mg/kg copper. The number of sniffs and a number of approaches towards Cu II males was significantly lower than towards control individuals. Also, in preference test with 150 mg/kg treated animals, total activity was lower towards copper treated animals. At the same time, copper did not influence intra-sexual interactions.

## Introduction

One of the most serious problems of the rapid industrial development is contamination by physiological substances occurring at excessive levels. Plants growing on contaminated soil may accumulate substantial concentrations of different metals, and then these plants may be ingested by herbivores (Wijnhoven et al. 2007). For animals living in polluted areas, often the only sources of water are contaminated. Significant concentrations of different metals have been found in the tissues of such animals (Gdula-Argasinska et al. 2004; Martiniakova et al. 2012; Martiniakova et al. 2011; Nikolov et al. 2010; Ullah et al. 2014; Wijnhoven et al. 2007; Zounkova et al. 2014). At the same time, in industrial districts a decline in the density of rodent populations has been widely observed (Kataev et al. 1994; Sheffield et al. 2001). There is no overall data available explaining this phenomenon. One of the reasons may be the altered reproduction of small rodents. Our previous studies have shown that excess of copper, a physiological trace element which, in extreme concentrations, may lead to degenerative changes in an organism (Brewer 2007; Brewer 2010), negatively influence reproductive abilities of small rodent males mainly by declining their sperm quality and quantity (Miska-Schramm et al. 2014). Therefore, it may potentially impact their sexual behavior. This, in combination with reproductive morphological parameters, influences reproductive success of the population. The model species in our experiment is the bank vole (*Myodes glareolus*, Schreber 1780). This animal was selected for several reasons.

The bank vole is thought to be the most common rodent species in Europe and Asia. It is a polyphagous species showing high plasticity in dietary requirements and undergoing large changes in diet during the annual cycle (Macdonald 2001). Generally the bank vole feeds mostly on herbs and tree seeds; it also supplements its diet with invertebrates (Eccard and Ylönen 2001; Eccard and Ylönen 2006). Plants growing on polluted sites, and the invertebrates living there, may accumulate increased amounts of copper (Chakroun et al. 2010; Lukáň 2009; Owojori and Siciliano 2012; Sękara et al. 2005). Organisms inhabiting industrial districts are exposed thorough their diet not only to metal-contaminated plants and invertebrates but also to contaminated water (Youngers et al. 2002).

For many years the bank vole was considered a polygynous species (Gipps 1985), but molecular techniques have revealed that females commonly mate with multiple males (Borkowska and Ratkiewicz 2010; Ratkiewicz and Borkowska 2000) and therefore should be considered promiscuous. Bank vole communication is based mainly on olfactory and auditory signals. The olfactory signals are highly volatile substances which can be used for long-distance communication. In the communication between conspecifics, the olfactory system plays roles in social interaction as well, as in the reproductive activity of bank voles (Marchlewska-Koj 2000). Pheromones are excreted mainly in urine and faeces, and are secreted through skin glands (Bind et al. 2013; Cheung 2008; Kannan and Archunan 2001). In sexual behavior they play an important role in enabling females to distinguish a potential mating partner, and in identifying social status or kinship (Kruczek 2007; Kruczek and Pochroń 1997). For example, rat females prefer males with stronger testosterone-controlled signals, such as male chemosignals (Xiao et al. 2004). Females show an increase of behavioral activity in response to chemosignals from dominant males (Horne and Ylönen 1996; Kruczek and Pochroń 1997), which have been shown to have better reproductive morphological parameters than subordinate ones (Kruczek and Styrna 2009).

In addition to using olfactory signals, bank voles also communicate by means of calls in two ranges: audible to humans (below 8 kHz) and high-frequency (ca. 20–35 kHz) (Kapusta et al. 2007; Marchlewska-Koj 2000). In nonsocial situations, the bank vole uses calls only when trapped or handled (Marchlewska-Koj 2000). In social behavior, the calls are associated with aggressive behavior during intra-male encounters (Kapusta and Pochroń 2011), sexual behavior in heterosexual interaction (Kapusta and Sales 2009) and infant-mother communication (Szentgyorgyi et al. 2008). In male-female encounters, bank vole males emit ultrasounds at 30–35 kHz, depending on the male’s earlier sexual experience (Kapusta et al. 1994; Marchlewska-Koj 2000). In the presence of females, sexually experienced males emit more signals than inexperienced ones (Kapusta et al. 1994). As has been shown in mice, females present a clear preference for vocalizing males over devocalized ones (Asaba et al. 2014; Hammerschmidt et al. 2009). Moreover, the response of females to male calling is clearly hormone-dependent: ovariectomized house mice show no preference for vocalizing males (Pomerantz et al. 1983). Vocal displays associated with sexual encounters are often mediated by testosterone released from the testes (Bass and Remage-Healey 2008; James et al. 2008; Pasch et al. 2011).

Copper of both natural and anthropogenic origin is present in natural ecosystems (Spatari et al. 2002). In reproductive biology, copper plays an important role as a component of enzymes with activity often correlated with endocrine function, for example, in the regulation of progesterone production by luteal cells via the involvement of superoxide dismutase (Sales et al. 2011), or regulation of the production of steroid hormone by copper via inhibition of the testicular enzyme 17beta-hydroxysteroid dehydrogenaze (Chattopadhyay et al. 2005). Molecular research has shown that copper can bind with estrogen receptor-α (ER -α) in human breast cancer cell line MCF-7 (Martin et al. 2003). This suggests that excess copper might also bind with ER-α in animal organisms. In human males, copper has been found to be positively associated with testosterone, and a positive relation between the levels of testosterone and LH has also been described (Meeker et al. 2010). Copper is also associated with a nonmonotonic decrease of thyroid-stimulating hormone (TSH), which can have adverse effects on development, behavior, metabolism, reproduction and other functions (Meeker et al. 2009). Ng and Liu (1990) suggested a direct toxic action of copper on steroid-producing cells in the adrenal gland and testes.

According to previous study, in the bank vole, copper inhibited spermatogenic activity, as indicated by a decrease of the spermatogenic index (Miska-Schramm et al. 2014). Oral administration of copper lowered bank vole sperm quality and quantity. This was expressed by higher proportions of sperm cells with abnormal heads, lower proportions of sperm cells with correct structure of the tail cytoplasmic membrane, and also with the lower proportions of viable and mobile sperm cells (Miska-Schramm et al. 2014). That experiment used two doses of copper: basal (150 mg/kg) and elevated (600 mg/kg). Both copper doses led to lower epididymal sperm number, but only the higher dose reduced the other sperm cell parameters; the lower copper dose also increased bank vole testes weight (Miska-Schramm et al. 2014). These findings suggested that the lower dose may have accelerated selected endocrine pathways (e.g., ER-α), while the higher dose was toxic to the bank vole males and harmed their reproductive parameters.

The data on the effects of excess copper on rodent females’ reproductive abilities is sparse, in contrast to the large amount of research on copper deficiency in this sex (Bureau et al. 2003; Keen et al. 2003). As mentioned above, it is thought that copper in females may act through neurohormonal pathways at the level of the hypothalamus (Michaluk and Kochman 2007). In the bank vole, copper did not affect the number of matured ovarian follicles; a dose of 600 mg/kg reduced female body weight, while 150 mg/kg increased relative uterus weight (Miska-Schramm et al. 2014). In view of these reproduction-related morphological disorders associated with copper, it is reasonable to suggest that copper might also modify rodents’ sexual behavior. To our knowledge, only two studies have been done to test this conjecture in vertebrates: in rats, ingesting copper-enriched food led to decreased aggression levels against same-sex individuals, and it suppressed male sexual behavior in the presence of rat females (Bataineh et al. 1998; Chattopadhyay et al. 1999). Therefore, in this paper, we have addressed the question: how do the environmentally available doses of copper (150 and 600 mg/kg) influence the sexual behavior of bank voles?

## Methods

### Animals and housing conditions

The bank voles (*Myodes glareolus*, Schreber 1780) came from the laboratory colony of the Institute of Environmental Sciences, Jagiellonian University, Krakow. The original stock was obtained in 1976 from the Mammal Research Institute of the Polish Academy of Sciences (Białowieża) and is maintained as an outbred stock colony, according to the system described by Green (1966). Briefly, each generation consists of at least 22 breeding pairs; the male and female of each mating pair do not share parents or grandparents. This breeding system ensures the heterogeneity of the colony (Green 1966). The animals are housed in polyethylene cages (40 cm × 25 cm × 15 cm) under a 14 h photoperiod (7 a.m.–9 p.m. light, 9 p.m.–7 a.m. dark) at 21 ± 1 °C and 60% humidity. Wood shavings are provided as bedding material and changed once a week. Standard pelleted chow for laboratory rodents (Labofeed H, Kcynia) and liquid in the form of deionized water or solutions of copper are available *ad libitum*.

For the study, at 19–20 days of age the weanlings were separated from their parents and placed in clean cages. At 4 weeks of age, 3–5 individuals were placed in same-sex cages. Then both females and males were randomly divided into three experimental groups. Starting from 4 weeks of age until the end of the experiments, the animals were treated with different metal solutions or given deionized water for 12 weeks.

### Experimental groups

C—control group given deionized water.

Cu I—(150 mg/kg dose): copper sulphate (II) 5 hydrate (CuSO_4_*5H_2_O) AR purity grade (AVANTOR, Poland) at concentration of 150 mg Cu^2+^/l (75 mg Cu^2+^/kg body weight/day).

Cu II—(600 mg/kg dose): copper sulphate (II) 5 hydrate (CuSO_4_*5H_2_O) AR purity grade (AVANTOR, Poland) at concentration of 600 mg Cu^2+^/l (300 mg Cu^2+^/kg body weight/day).

### Effect of copper on bank vole behavior

2 weeks before the behavior tests, each 14-week-old individual was placed in a separate cage. Individuals of similar body weight were chosen for each behavioral experiment. On the day of the test the animals were transferred with their home cages to a bio-acoustic chamber for 1 h for habituation prior to testing.

### Male-female interactions

The behavior and vocalization of male-female pairs were recorded during open-field tests in the following combinations: ♂C vs. ♀C; ♂Cu I vs. ♀C; ♂Cu II vs. ♀C. After habituation, the female and male were transferred from their home cages to a glass vivarium (40 × 20 × 25 cm) with the floor covered with clean wood shavings. The vivarium was divided by a metal partition into two halves, each occupied by one animal, and left undisturbed for 5 min prior to the test. After this period the partition was removed, and the behavior and vocalizations were recorded for 10 min. Observations were made between 8 a.m. and noon.

Behavior was recorded with a Sony DCR-HC30E digital camera. Sounds were recorded with an ultrasound microphone suspended 10 cm above the tested animals. The ultrasound microphone was connected to a QMC (UK) type S30 ultrasound detector coupled to a Sony (Japan) MZ-RH10 minidisk recorder. The behavior and sound recordings were started and terminated simultaneously. Twelve pairs from each of the aforementioned combinations, in which the animals approached each other during the 10-min testing period were further analyzed.

The behavior of males and females during the 10-min sessions was analyzed using a VLC Media Player.

Amicable behavior was assessed as:

– latency of nonaggressive approach (in sec) after removal of the partition; first physical contact or sniffing at distance less than 1 cm

– number of nonaggressive approaches

– time spent sniffing (in sec)

– self-grooming (in sec)

– environment investigation (in sec).

Aggressive behavior was assessed as:

– latency of attack (in sec) after removal of the partition

– number of aggressive approaches, boxing and wrestling

– time spent fighting (in sec).

“Approach” was defined as directional locomotion toward the other animal. A “sniff” was recorded when the individual directed its nose to within ca. 0.5 cm of the other individual. “Total activity” was taken as the sum of approaches and sniffs toward the other animal during the test.

The recorded signals were copied from the minidisc to a computer and analyzed using SpectraPLUS ver. 2.32 (Sound Technology Inc., Campbell, USA). Latency (in sec) and the number of emitted ultrasounds in the 10-min recording were noted.

### Female sexual preference

Tests were performed in a glass vivarium divided into three equal compartments with the floor covered with clean wood shavings. The vivarium was changed to a clean one before each test. As described above, the females were transferred with their home cages to a bio-acoustic chamber for habituation for 1 h prior to testing. Then, 5 min before recording, the female was transferred from the home cage to the middle section of a vivarium. Two males anaesthetized with Vetbutal (0.1 ml/10 g body weight; Biowet, Puławy, Poland) were placed in each of the two side compartments and the partitions were removed. The males were anaesthetized due to concern that their variable behavior would complicate the task of interpreting the behavioral response of the females. Female behavior was recorded in the following combinations: ♀C vs. ♂C & ♂Cu I; ♀C vs. ♂C & ♂Cu II; namely, each female was exposed to control and Cu experimental group males (either Cu I or Cu II) located randomly in the vivarium. The behavior of the 12 females from each of these combinations was taped with a Sony DCR-HC30E digital video camera for 10 min, beginning as soon as the female had sniffed each male once. The number of female-to-male approaches, the number of sniffs, and the time spent sniffing each male were measured using VLC Media Player software. Approaches and sniffs were defined as described above.

The researchers who performed and analyzed the behavioral tests were blind to the experimental group during those procedures.

### Statistical analysis

The following statistical tests were used to analyze the data:

– Kruskal-Wallis one-way test for nonparametric analysis: for latency of nonaggressive approach, aggressive approach and ultrasound emission; for time of sniffs, attacks, environmental activity and self-grooming;

– One way ANOVA: number of sniffs, attacks, nonaggressive and aggressive approaches, ultrasounds;

– Post-hoc Tukey test following one way ANOVA, and Mann-Whitney U test following Kruskal-Wallis one way test: to test the significance of differences between means;

– Wilcoxon matched-pairs test for dependent samples in preference tests; for number of approaches, number and duration of sniffs, total activity.

All procedures employed STATISTICA v. 10. All data are presented as mean ± SE. The level of statistical significance was deemed to be *p* < 0.05.

## Results

### Male-female interactions

Males and female behavior in heterosexual interaction is presented in Table 1. Copper did not affect any intrasexual behavior. There were no significant differences in latency to first approach [sec] or number of aggressive approaches and nonaggressive approaches between animals from the tested combinations (♂C vs. ♀C; ♂Cu I vs. ♀C; ♂Cu II vs. ♀C). Similarly, there were no significant differences in latency to first attack [sec], number of attacks, sniffing time [sec] or number of sniffs (Table 1) between the tested pairs. As presented in Table 1, the number of ultrasonic calls did not differ with treatment, and there were no significant differences in latency to first ultrasound [sec] between animals from the tested combinations. All tested male-female pairs of bank voles emitted ultrasounds at a mean frequency of 27–33 kHz. Table 2a presents female behavior towards males from the treatment groups (C, Cu I, Cu II). There were no significant differences in number of female approaches (aggressive; nonaggressive) to males from the different experimental groups. No significant differences in females’ sniffing behavior were found between animals from the tested pairs (number of sniffs; sniffing time [sec]). Similarly, no significant differences were found in female time spent on environmental activity [sec] or self-grooming [sec] (Table 2a) between animals from the different experimental combinations. Table 2b presents the results for the behavior of males given water or the copper solutions (Cu I, Cu II) towards control females. There were no significant differences in the number of male approaches to control females (aggressive; nonaggressive; Table 2b) or in male sniffing behavior (number of sniffs; sniffing time) between animals from the tested pairs. Similarly to female behavior, there were no significant differences in male environmental activity [sec] or self-grooming [sec] (Table 2b) between the animals of the experimental groups.

**Table 1:** Latency to first approach, attack, ultrasonic call; number of aggressive approaches, nonaggressive approaches, sniffs, ultrasonic calls; time of sniffing and attacks presented by control females (C) and by water or copper-treated males (C, Cu I or Cu II) in 10-min open-field test

	♀C vs. ♂C	♀C vs. ♂Cu I	♀C vs. ♂Cu II		*p*
Latency to first approach [sec]	77.3 ± 19.5	70.1 ± 14.8	84.9 ± 26.8	H_(2,33)_ = 0.01	NS
Aggressive approaches [no.]	6.7 ± 1.4	5.8 ± 1.2	4.8 ± 1.0	F_(2,33)_ = 0.55	NS
Nonaggressive approaches [no.]	7.3 ± 2.4	6.9 ± 2.2	6.2 ± 1.2	F_(2,33)_ = 0.07	NS
Latency to first attack [sec]	147.2 ± 46.9	197.1 ± 71.3	240.9 ± 70.9	H_(2,33)_ = 0.18	NS
Attack time [sec]	22.0 ± 5.8	13.9 ± 3.4	10.5 ± 3.1	H_(2,33)_ = 2.42	NS
Sniffs [no]	19.3 ± 6.2	17.6 ± 3.7	22.8 ± 4.6	F_(2,33)_ = 0.29	NS
Sniffing time [sec]	99.6 ± 26.6	96.3 ± 21.5	170.5 ± 36.7	H_(2,33)_ = 2.63	NS
Latency to first ultrasound [sec]	89.3 ± 25.0	63.1 ± 20.4	67.8 ± 18.0	H_(2,33)_ = 0.82	NS
Ultrasonic calls [no.]	116.3 ± 17.5	110.6 ± 37.8	120.9 ± 18.2	F_(2,33)_ = 0.96	NS

Means ± S.E

**Table 2:** Latency to first approach, attack, ultrasonic call; number of aggressive approaches, nonaggressive approaches, sniffs, ultrasonic calls; time of sniffing and attacks presented by (a) control female (C) in interaction with male (C, Cu I, Cu II) or by (b) male (C, Cu I, Cu II) in interaction with control female in 10-min open-field test

	♀C vs. ♂C	♀C vs. ♂Cu I	♀C vs. ♂Cu II		*p*
(a)					
Aggressive approaches [no.]	3.2 ± 0.7	3 ± 0.3	2.4 ± 0.5	F_(2,33)_ = 0.66	NS
Nonaggressive approaches [no.]	2.9 ± 1.4	2.7 ± 0.9	2.3 ± 2.5	F_(2,33)_ = 0.92	NS
Sniffs [no.]	16.6 ± 8.4	6.9 ± 1.9	8.6 ± 7.1	F_(2,33)_ = 0.35	NS
Sniffing time [sec]	32.8 ± 11.7	37.1 ± 14.8	51.7 ± 15.0	H_(2,33)_ = 0.91	NS
Environmental activity [sec]	108.4 ± 30.3	93.9 ± 27.6	143.4 ± 27.1	H_(2,33)_ = 1.92	NS
Self-grooming [sec]	19.4 ± 7.5	9.7 ± 3.5	14.9 ± 4.6	H_(2,33)_ = 1.11	NS
(b)					
Aggressive approaches [no.]	3.2 ± 0.7	2.8 ± 0.6	2.4 ± 0.5	F_(2,33)_ = 0.7	NS
Nonaggressive approaches [no.]	4.3 ± 1.4	4.3 ± 1.9	3.8 ± 1.0	F_(2,33)_ = 0.97	NS
Sniffs [no.]	10.6 ± 3.7	10.7 ± 2.9	14.3 ± 3.4	F_(2,33)_ = 0.68	NS
Sniffing time [sec]	58.8 ± 20.0	59.3 ± 15.2	103.9 ± 23.4	H_(2,33)_ = 2.45	NS
Environmental activity [sec]	94.4 ± 21.2	84.1 ± 19.3	149.0 ± 28.3	H_(2,33)_ = 3.47	NS
Self-grooming [sec]	14.5 ± 4.7	7.5 ± 3.2	12.1 ± 2.8	H_(2,33)_ = 2.1	NS

Means ± S.E

### Female sexual preference

**Fig. 1:**
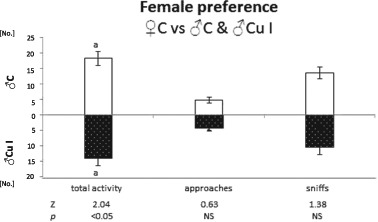
Total activity, number of approaches and sniffs presented by control female towards anaesthetized male treated with 150 mg/kg copper solution (Cu I; pattern bars) and control male (C; open bars). Means bearing the same letter differ significantly; a—*p* < 0.05. Number of tested females —12. Means ± S.E

**Fig. 2:**
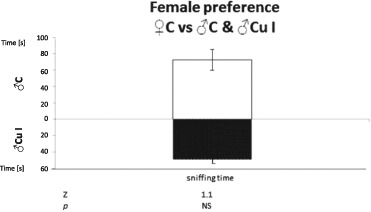
Time of sniffing presented by control female towards anaesthetized male treated with 150 mg/kg copper solution (Cu I; pattern bars) and control male (C; open bars). Number of tested females—12. Means ± S.E

Control female behavior towards control (C) males and those treated with 150 mg/kg copper (Cu I) is summarized in Figs. 1 and 2. Control females presented significantly higher total activity (number of approaches + number of sniffs) toward control males (${\bar{\mathrm X}}$ = 18.33 ± 2.25) than toward males treated with 150 mg/kg copper (${\bar{\mathrm X}}$ = 14.08 ± 2.48; *p* < 0.05). However, there were no significant differences in the number of female approaches to control males (${\bar{\mathrm X}}$ = 4.75 ± 0.99) and those treated with 150 mg/kg copper (${\bar{\mathrm X}}$ = 4.33 ± 0.93), nor in the number of females’ sniffs of control (${\bar{\mathrm X}}$ = 13.58 ± 1.88) and Cu I males (${\bar{\mathrm X}}$ = 10.58 ± 2.23) (Fig. 1). Similarly, there were no significant differences in control females’ sniffing time [sec] toward control (${\bar{\mathrm X}}$ = 72.50 ± 12.82) and Cu I males (${\bar{\mathrm X}}$ = 49.00 ± 11.82).

**Fig. 3:**
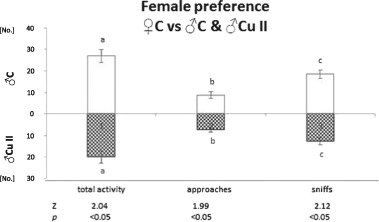
Total activity, number of approaches and sniffs presented by control female towards anaesthetized male treated with 600 mg/kg copper solution (Cu II; pattern bars) and control male (C; open bars); Means bearing the same letter differ significantly; a, b, c—*p* < 0.05. Number of tested females—12. Means ± S.E

**Fig. 4:**
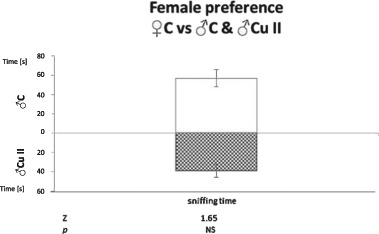
Time of sniffing presented by control female towards anaesthetized male treated with 600 mg/kg copper solution (Cu II; pattern bar) and control male (C; open bar). Number of tested females—12. Means ± S.E

The results given in Figs. 3 and 4 show that females preferred control males to those treated with the higher copper dose (Cu II), as shown by higher total activity towards control males (${\bar{\mathrm X}}$ = 26.92 ± 2.94) than toward Cu II males (${\bar{\mathrm X}}$ = 20.0 ± 2.77; *p* < 0.05), higher number of female approaches to control males (${\bar{\mathrm X}}$ = 8.67 ± 1.54) than to Cu II males (${\bar{\mathrm X}}$ = 7.25 ± 1.15; *p* < 0.05), and higher number of sniffs of control males (${\bar{\mathrm X}}$ = 18.5 ± 1.96) than Cu II males (${\bar{\mathrm X}}$ = 12.75 ± 1.76; *p* < 0.05). However, there were no significant differences in sniffing time [sec] presented by control females towards C males (${\bar{\mathrm X}}$ = 56.71 ± 8.87) and Cu II males (${\bar{\mathrm X}}$ = 39.15 ± 6.81).

## Discussion

The vast majority of publications concerning the influence of copper on rodents’ behavior present findings on rodent models of human diseases. So-called toxic milk mice as well as Long-Evans cinnamon rats are animal models for Wilson disease (Theophilos et al. 1996; Yoshida et al. 1987). A genetic disorder of copper metabolism occurs in those animal models as well as in human patients with Wilson disease (Ala et al. 2007; Allen et al. 2006). In those individuals, copper accumulates mainly in the liver and the brain; consequently, liver disease and neuropsychiatric symptoms are the main features that lead to a diagnosis (Ala et al. 2007). Behavioral disturbances have been observed in toxic milk mice and in Long-Evans cinnamon rats (Fujiwara et al. 2006; Przybyłkowski et al. 2013; Terwel et al. 2011).

In the context of findings on copper-induced hormonal disorders, sexual behavior might be expected to be affected by copper exposure. For this reason, our study included behavioral tests of male-female interactions. At both tested concentrations, copper did not influence bank vole social or aggressive behavior, in contrast to results from other research. In rats, oral administration of 200 mg/kg copper sulphate, a dose similar to the lower dose in our experiments, led to neurobehavioral abnormalities manifested in reduced latency to fall in a rotarod and lower attention percentage scores (Kumar et al. 2015). Long-term ingestion of copper chloride suppressed the sexual behavior of male rats (Bataineh et al. 1998), but that effect occurred after exposure to a much higher Cu dose (1000 mg/kg) than used in this study. Our experiments used copper doses equivalent to environmental levels found in polluted districts. The results of this work may better represent the effects of normally presented copper concentrations in polluted areas.

One element of sexual behavior is sexual selection. In bank voles, the females choose the sexual partner, while the males attract females using olfactory and auditory cues (Gipps 1985; Kapusta 2012; Kruczek 2007; Kruczek and Pochroń 1997). Production, excretion and emission of olfactory and auditory signals might be expected to be modified by copper. In our behavioral experiment the males were given copper in solution and the females were given only deionized water. We chose to use open-field two-choice preference tests to examine female sexual preference, because all research of the Mammalian Research Group has employed the same methodology, making it easy to compare results. In these tests, control females were subjected to two anesthetized males of similar size, so their choice would be based mainly on olfactory cues. The females showed a clear preference for control males when subjected to a control and a male treated with 600 mg/kg copper. Exposure to 600 mg/kg copper decreased male sexual attractiveness, probably due to disorders of olfactory cue production and excretion.

In the wild, reproductive success requires social, aggressive and sexual behavior. Based on our results, it is not possible to state that copper interferes with social and aggressive behavior, but the findings show that a high level of copper definitely interferes with olfactory cue production. Copper impairs lower vertebrates’ chemical communication abilities and behavior, including sexual behavior (Lahman et al. 2015). There is no published data on rodent females’ sexual preference for particular males after chronic exposure to copper. Copper may become an oxidative stress factor; in mice this stress may reduce male investment in the molecular components of olfactory signaling essential for mate attraction (Garratt et al. 2014). This mechanism might also operate in the bank vole; it would explain the females’ loss of interest in males treated with 600 mg/kg copper.
